# Global prevalence of obesity in the older adults: A meta-analysis

**DOI:** 10.1016/j.puhip.2025.100585

**Published:** 2025-01-18

**Authors:** Ali Asghar Khaleghi, Nader Salari, Niloofar Darvishi, Shadi Bokaee, Samira Jafari, Mahvan Hemmati, Masoud Mohammadi

**Affiliations:** a Department of Emergency Medicine, Faculty of Medicine, Fasa University of Medical Sciences and Health Services, Fars, Iran; b Department of Biostatistics, School of Health, Kermanshah University of Medical Sciences, Kermanshah, Iran; c Department of Psychiatric Nursing, Faculty of Nursing School, Tehran Medical Sciences, Islamic Azad University Science and Research Branch, Tehran, Iran; d Faculty of Health and Life Sciences, School of Life Sciences, Coventry University, Coventry, UK; e Student Research Committee, Kermanshah University of Medical Sciences, Kermanshah, Iran; f Research Center for Social Determinants of Health, Jahrom University of Medical Sciences, Jahrom, Iran

**Keywords:** Prevalence, Older adults, Obesity, Meta-analysis

## Abstract

**Objective:**

Obesity is a chronic and complex disease defined as the excessive accumulation of body fat and is one of the leading public health problems in developed and developing countries. Due to the importance of obesity, this study was conducted to investigate the prevalence of obesity in the older adults.

**Study design:**

meta-analysis.

**Methods:**

In this study, systematic review and meta-analysis of study data on the prevalence of obesity in the older adults in the world using keywords including: prevalence, outbreak, Body Mass Index, BMI, obesity, Elderly, aged, older adult, in Science Direct databases, Scopus, PubMed, Web of Science, Iran Doc, Mag Iran, SID and Google Scholar search engine were extracted without time limit until August 2020. The target population under study is the world's elderly, and obesity means a BMI≥30. The Random Effects Model was used to perform the analysis and, Comprehensive Meta-Analysis Software version 2.0 was used for data analysis.

**Results:**

In review 44 studies with a total sample size of 45,745,944 prevalence of obesity in the older adults of the world; In a meta-analysis of 25.3 % (95 % CI: 21.9–29). It was found that the highest prevalence of obesity in the older adults in South America with 40.4 % (95 % CI: 12.5–76.4). In addition, continental Europe with 33.6 % (95 % confidence interval: 24.1–44.5). The meta-regression results showed an increasing trend in the prevalence of obesity in the older adults in the world with an increasing sample size and a decreasing trend with increasing the study (P < 0.05).

**Conclusion:**

Given that the prevalence of obesity in the older adults is high, health policymakers must take adequate measures to increase public awareness about the risks of obesity in the older adults.

## 1 Background

Obesity is a chronic and complex disease that is defined as the excessive accumulation of body fat. Obesity is one of the leading public health problems in developed countries and developing countries [1,2]. In the 21st century, obesity has become one of the biggest health challenges in the world [3]. Increased body fat leads to an increase in total body mass in women and men [4]. Therefore, body weight is generally used as an indicator to diagnose obesity [4]. In most epidemiological studies on obesity, the body mass index is usually used to classify individuals in the overweight or obesity group [5]. Body mass index (BMI) is simply calculated from the ratio of body weight (kg) to height squared (meters) [4]. Other methods for assessing obesity include measuring waist circumference (WC), waist-to-hip ratio (WHR), waist-to-height ratio (WHtR), skinfold thickness, hydrostatic densitometry, and energy absorption. X-ray (DEXA) noted [4].

Increased obesity prevalence has been observed in all age groups, ethnic groups, men and women, and in all levels of education [6]. Accumulation of excess fat in men; In the trunk and abdomen, it is more common in women around the thighs and buttocks [7]. In recent decades, children, adolescents, and the elderly in many countries (including in recent years in middle- and low-income countries) have reported high rates of overweight and obesity [8], which has many consequences for people in childhood and brings with its adulthood [9]. The prevalence of obesity increases with age and exposes the elderly to dangerous diseases. It is noteworthy that this complication's majority in the elderly is increasing [10,11].

In different articles, the term “elderly” is defined in different ways. In some texts, “old” men and women are defined as people between 65 and 100 years old. In Harris's study, “elderly” refers to people with an average age of 77 years [12]. Another Italian group study looked at “elderly” people between the ages of 67 and 78 [13]. In contrast, another study [14] looked at " elderly women with obese” in a group with an average age refers to 58 years [15]. People over the age of 65 are usually considered older adults, regarded as an essential anthropological phenomenon. According to forecasts, the elderly population will increase from 600 million in 2000 to 1.2 billion, twice as much in 2025 [16].

The effects of overweight and obesity on disease and mortality have been known for more than 2000 years, with overweight and obesity accounting for 5 % of world mortality and causing 2.8 million deaths annually [17,18]. Mortality from obesity is expected to be higher in the future than smoking [19]. Obesity aggravates and causes many diseases in people. Such conditions can include diseases such as type 2 diabetes, hypertension, dyslipidaemia, ischemic heart disease, obstructive sleep apnoea, asthma, non-alcoholic osteo hepatitis, acid reflux disease of the oesophagus, degenerative joint disease of the back, buttocks, Knees, and legs, infertility, and polycystic ovary syndrome, various malignancies and depression [19].

Various factors increase the prevalence of obesity in the elderly, such as inactivity, poor eating habits, basal metabolism, and decreased nutritional needs. In the past, obesity was considered a “secondary” pathology that was not medically significant. However, today, obesity is increasingly being studied in the elderly because it causes disability and quality of life [20]. Obesity in the elderly has other negative consequences because it reduces physical activity and increases the nursing home's admission rate. Obesity may be the most significant cause of disability in the elderly [21].

According to published reports, about two-thirds of the world's adults were affected by obesity or overweight [1]. In 2005, the total number of affected by overweight adults in the world was 937 million, and the number of people affected by obese was 396 million [3]. Epidemiological studies show that Obesity increases the risk of breast, colon, colon, and cervical cancers, and 15–45 % of these cancers are related to obesity. The National Cancer Institute in the United States reports that breast cancer is twice as common in people with obese as in ordinary people [1].

Considering that the studies conducted in the field of obesity in the older adults in different countries have reported different results, this study aims to reduce the heterogeneity of these results and report the overall prevalence with a comprehensive review of all databases. Given the importance of what has been said and the growing trend of obesity in the elderly, the purpose of this study is to review the articles and estimate the prevalence of obesity in the elderly in the world. Due to the association of obesity with some aspects of mental health, including social functioning, physical function, anxiety and mental health, and little knowledge about the health of the elderly, officials and experts must be aware of this problem and its various dimensions. By doing this study, we can help the health system policymakers to make effective interventions.

## 2 Methods

In this systematic review and meta-analysis to find related studies, the databases of Science Direct, Scopus, PubMed, Web of Science (WoS), Iran Doc, Mag Iran, SID, and the Google Scholar search engine were searched. To achieve the desired articles, using the keywords, including prevalence, outbreak, Body Mass Index, BMI, Obesity, Elderly, aged, older adult, and all possible combinations of these words, the search strategy for each of the databases was determined. There was no time limit in the search process, so all possible related articles published by August 2020 were identified, and their information was transferred to the EndNote. To maximize the comprehensiveness of the search, the list of references used in all relevant articles found in the above search was manually reviewed. In this study, people with a BMI above 30 were considered obese [[22], [23], [24], [25], [26], [27]].

Our study aimed to answer the following research question " What is the global prevalence of obesity in the older adults?” According to the study population (Population) includes: The older adult's population over 60 years old based on studies, Outcome include: Prevalence of obesity means a BMI≥30, and study type (study design) includes: cross sectional studies.

## 3 Inclusion criteria

Inclusion criteria include: (1) cross-sectional studies, (2) studies that examined the prevalence of obesity in the older adults, (3) studies that have full text, (4) high and medium quality studies (score 16 and Above that), (5) studies that have undergone the judging process and have been accepted until the implementation of the systematic review process in this study (August 25th, 2020). (6) Studies that considered obesity (BMI≥30).

## 4 Exit criteria

Case-control and cohort studies, (2) case series, (3) case report, (4) review studies, (5) studies whose full text is not available, (6) Letter to the editor, (7)) Studies that have not undergone the judging process, and (8) low-quality studies and quality scores less than 16, (9) studies whose working method was not exact, and (10) other criteria for assessing obesity except (BMI) ≥30).

## 5 Quality assessment and evaluation of the risk of bias

The Newcastle-Ottawa Scale (NOS) is a quality assessment tool for this study. The NOS assigns up to a maximum of nine points for the least risk of bias in three domains: four points for selection of study groups; two points for comparability of groups and three points for ascertainment of exposure and outcomes. Eventually, articles were classified as high quality (scoring ≥5 points) or low quality (scoring<5 points).

## 6 Selection of studies

Initially, duplicate studies in various searched databases were excluded from this study. Then, this study prepared a list of titles of all the remaining articles, to obtain qualified articles by evaluating the articles in this list. In the first stage, screening, the titles and abstracts of the remaining articles were carefully studied, and irrelevant articles were removed based on the inclusion and exclusion criteria. In the second stage, the evaluation of the suitability of the studies, the full text of the possible relevant articles remaining from the screening stage was examined based on the inclusion and exclusion criteria. In this stage, unrelated studies were eliminated. To avoid bias, two researchers independently of each other performed all steps of reviewing sources and extracting data. If the articles are not included, the reason for deleting them was mentioned. In cases where there was disagreement between the two researchers, a third party reviewed the article. A total of 44 studies entered the final stage.

## 7 Extracting the data

Information on all final papers entered into the systematic review and meta-analysis process was extracted from a pre-prepared checklist. This checklist included the name of the first author, year of publication, place of study (country), study (continent), sample size, mean sample age, obesity testing tool, and volume of people with obese.

## 8 Statistical analysis

Heterogeneity of studies was assessed using the I^2^ test. Publication bias was assessed the Begg and Mazumdar correlation test and corresponding Funnel Plots were drawn. Data were analysed within Comprehensive Meta-Analysis software (version 2). Due to the heterogeneity in the studies, the random effects model was used for meta-analysis, and the meta-regression method was used to investigate the factors affecting the heterogeneity.

## 9 Results

**Fig. 1:**
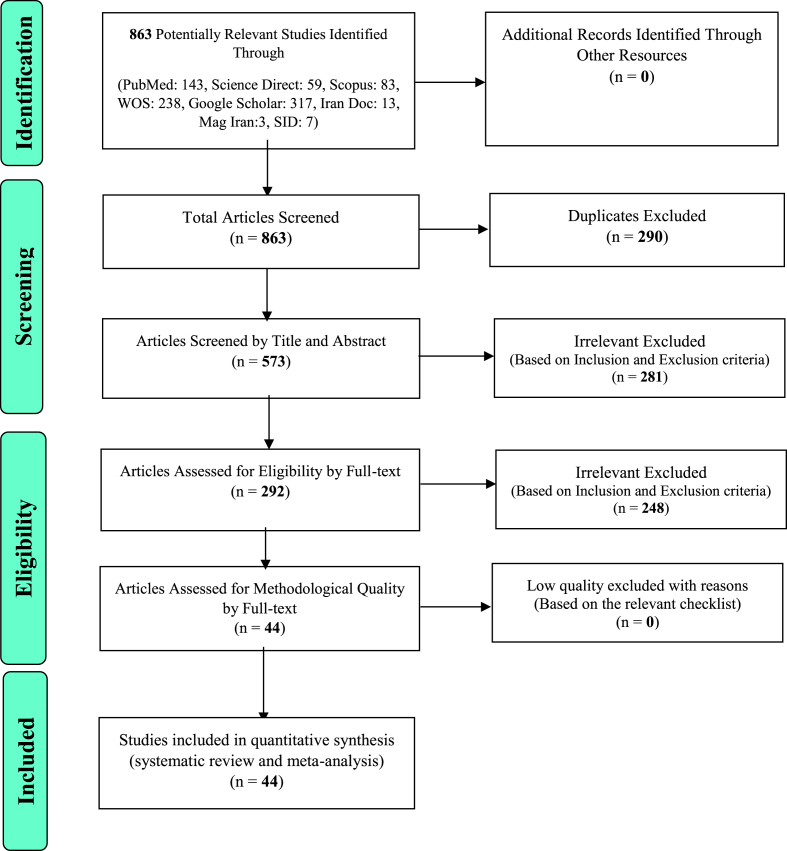
The flowchart on the stages of including the studies in the systematic review and meta-analysis (PRISMA 2009).

In this study, systematic review and meta-analysis of information on studies on the prevalence of obesity in the older adults in the world without time constraints and according to PRISMA guidelines were systematically reviewed. Based on the database's initial search, 863 possible related articles were identified and transferred to information management software (Endnote). Out of a total of 863 identified studies from the database, 290 duplicate studies were excluded. In the screening stage, out of 573 studies, 281 articles were removed by studying the title and abstract based on inclusion and exclusion criteria. In the competency evaluation stage, out of the remaining 292 studies, 248 articles were deleted by studying the article's full text based on the inclusion and exclusion criteria due to irrelevance and the lack of full text. Finally, 44 studies were reviewed (Fig. 1).

The results of a systematic review of studies in Table 1 were reported based on indicators of the prevalence of obesity in the older adults as well as the country in which the study was conducted. 2015) (104 people) and David E. Arterburn et al. (2004) (45,625,000 people). The characteristics of the eligible studies included in the meta-analysis are given in Table 1 (Table 1).

**Table 1:** Data related to the studied studies.

Sample number	First author	Publication year	Research location (country)	Research location (continent)	Total sample size	Quality assessment
1	Fabíola Bof de Andrade [28]	2012	Brazil	South America	833	Moderate
2	Javier Aranceta-Bartrin [29]	2016	Spain	Europe	3966	High
3	DavidE.Arterburn [30]	2004	America	North America	45,625,000	Moderate
4	M. Asp [31]	2017	Sweden	Europe	2409	High
5	J. Benito-Leon [32]	2013	Spain	Europe	1949	Moderate
6	Odilia I. Bermudez [33]	2001	Massachusetts	North America	835	Moderate
7	Diana P. Brostow [34]	2019	America	North America	2868	High
8	Anne O. Carter [35]	2006	Barbados	North America	1508	Moderate
9	Dara W. Ford [36]	2014	USA	North America	4009	Moderate
10	R. Gepstein [37]	2004	USA	North America	298	Moderate
11	Theresa E. Gildner [38]	2015	China	Asia	13,974	High
			India	Asia	10,915	High
			Mexico	North America	2438	High
			Russia	Europe	3893	High
			South Africa	Africa	4000	High
12	Alba Gómez-Cabello [39]	2012	Spain	Europe	3136	Moderate
13	A. Gomez-Cabello [40]	2011	Spain	Europe	3037	Moderate
14	S Goya Wannamethee [41]	2004	England	Europe	4232	Moderate
15	Hajian-Tilaki K [42]	2016	Iran	Asia	750	High
16	T S Han, D M Lee [43]	2015	Europe	Europe	3319	Moderate
17	A Jahangir [25]	2018	USA	North America	904	High
18	Pierre Jésus [27]	2017	Central Africa	Africa	990	Moderate
19	C. Kiss [44]	2003	Hungary	Europe	641	Moderate
20	Darius N Lakdawalla [24]	2005	USA	North America	2757	Moderate
21	Luısa Helena do Nascimento [45]	2013	Brazil	South America	900	Moderate
22	Lucas H. McCarthy [46]	2009	USA	North America	404	Moderate
23	Beatriz Moreno-Vecino [47]	2015	Spain	Europe	471	Moderate
24	M. Nematy [48]	2009	Iran	Asia	1962	Moderate
25	Orit Ofir [49]	2019	Israel	Asia	669	High
26	Wilna H Oldewage-Theron [50]	2015	South Africa	Africa	104	Moderate
27	Otitoola OC [51]	2015	South Africa	Africa	398	Moderate
28	A Planas [52]	2001	Spain	Europe	605	Moderate
29	Josep Redon [53]	2008	Spain	Europe	6263	Moderate
30	Ruchit Shah [54]	2015	USA	North America	675	High
31	Woo-Jung Song [55]	2012	Korea	Asia	994	High
32	Tommi Sulander [56]	2007	Finland	Europe	7482	Moderate
33	Suzana S, MMed Sci [57],	2012	Malaysia	Asia	4746	Moderate
34	Elizabeth Vásquez [58]	2014	USA	North America	5304	Moderate
35	Qian Wang [59]	2018	China	Asia	7070	High
36	Evelyn Wong [60]	2016	Australia	Oceania	1696	High
37	Jean Woo [61]	2007	China	Asia	4000	Moderate
38	Kyuhyun Yoon [62]	2014	Korea	Asia	510	Moderate
39	E Zoico [63]	2004	Italy	Europe	167	Moderate
40	Masoumeh Tawhidi [64]	2009	Iran	Asia	855	High
41	Dr. Reza Nouri [65]	2011	Iran	Asia	727	High
42	Andrzej Jaroszynski [66]	2016	Poland	Europe	887	High
43	Laith Thamer Al-Ameri [67]	2018	Baghdad	Asia	162	High
44	El Bcheraoui C [68]	2008	Lebanon	Asia	232	Moderate

**Fig. 2:**
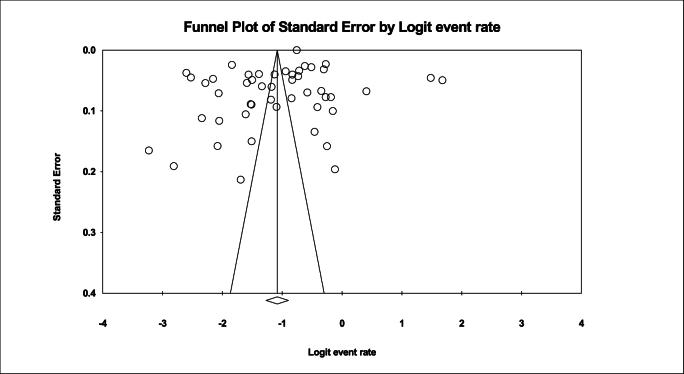
Funnel plot results on the prevalence of obesity in the older adults in the world.

**Fig. 3:**
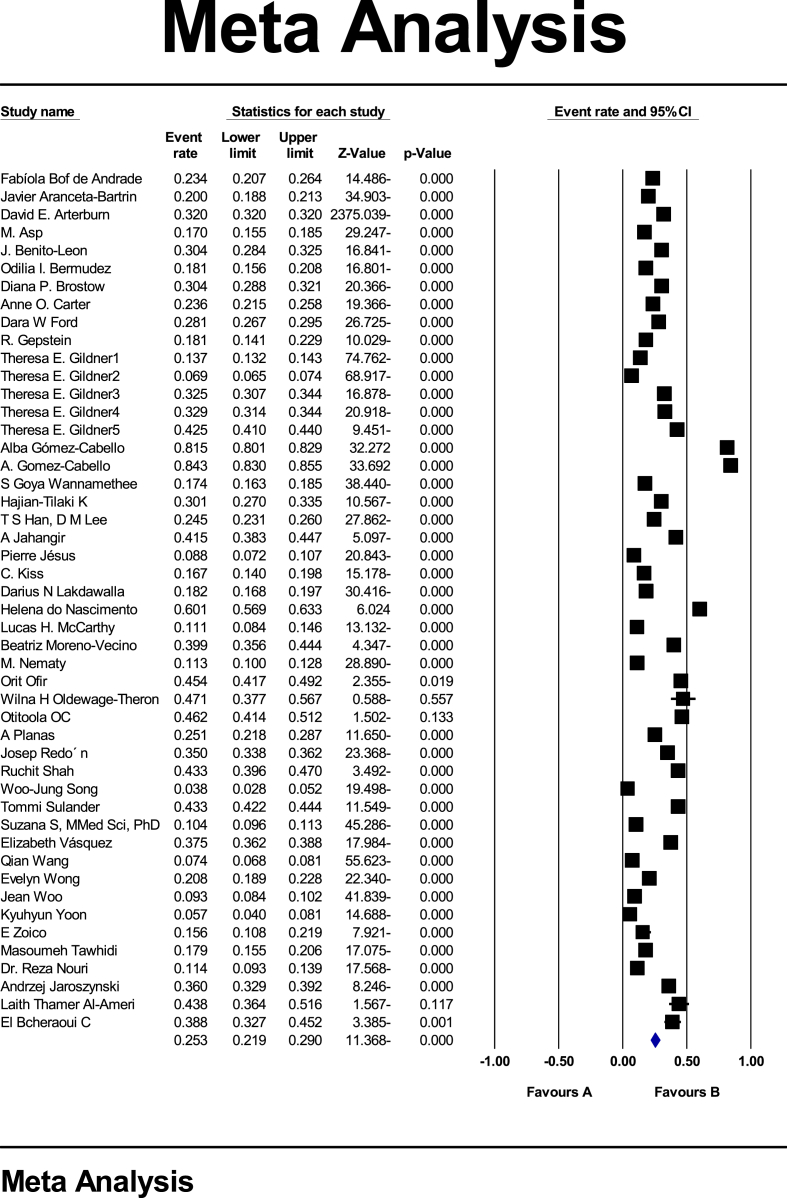
The prevalence of obesity in the older adults in worldwide and the 95 % confidence interval based on a random effect model.

Based on the test results (I^2^: 99.7) and due to the heterogeneity of selected studies, a random effects model was used to combine studies and shared prevalence estimates. The reason for heterogeneity between studies can be due to differences in sample size, sampling error, year of study or place of study. The probability of publication bias in this study by funnel diagram and Begg and Mazumdar correlation test at a significance level of 0.1 showed no publication bias of the prevalence in the present study (P: 0.728) (Fig. 2).In the study of 44 studies with a total sample size of 45,745,944 Prevalence of obesity in the older adults of the world; 25.3 % (95 % CI: 21.9–29) was obtained (Fig. 3).

## 10 Meta-regression test

**Fig. 4:**
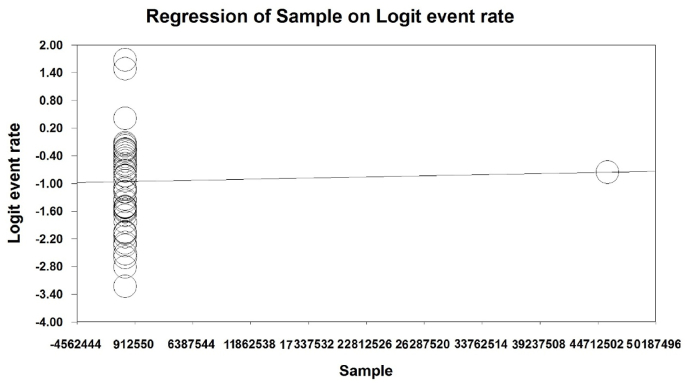
Meta regression chart of the prevalence of obesity in the older adults of the world by sample size.

**Fig. 5:**
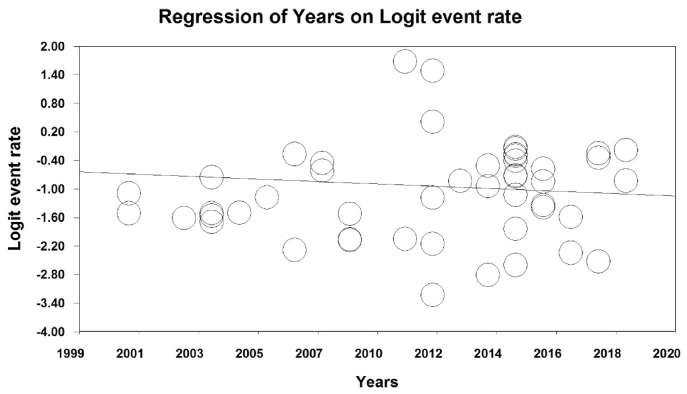
Meta-regression chart of the prevalence of obesity in the older adults of the world by year.

To investigate the effects of potential factors affecting the heterogeneity of the prevalence of obesity in the world's older adults, meta-regression was used for two factors of sample size, and year of study (Fig. 4, Fig. 5). According to Fig. 4, with increasing the sample size, the prevalence of obesity in the world's older adults increases, which is statistically significant (P < 0.05). and the prevalence of obesity in older adults decreases as the number of years of study increases, the difference was also statistically significant (P < 0.05) (Fig. 5).

## 11 Subgroup analysis

Table 2, which reports the prevalence of obesity in the older adults in the world by different continents, these changes are reported in Asia, Europe, Africa, North and South America and Oceania. The highest prevalence reported is in the South America continent with 40.4 % (95 % confidence interval: 12.4–76.4 and continental Europe with 33.6 % (95 % confidence interval: 24.1–44.5) and the lowest prevalence reported is in the Asia continent with 14.6 % (95 % confidence interval: 10.7–19.5 (Table 2).

**Table 2:** Prevalence of obesity in the older adults of the world by different continents.

Continents	Number of articles	Sample Size	I^2^	Prevalence % (95 % CI)
Asia	14	47,566	99.1	14.6 (95 % CI: 10.7–19.5)
Oceania	1	1696	100	20.8 (95 % CI: 18.9–22.8)
South America	2	1733	99.5	40.4 (95 % CI: 12.5–76.4)
North America	12	45,647,000	98.2	27.3 (95 % CI: 24.4–30.5)
Europe	15	42,457	99.7	33.6 (95 % CI: 24.1–44.5)
Africa	4	5492	99.04	32.5 (95 % CI: 16.1–54.8)

## 12 Discussion

In the present study, the prevalence of obesity in the older adults of the world; It was 25.3 % that the highest prevalence of obesity in the older adults in South America with 40.4 % and continental Europe with 33.6 %. The prevalence of obesity in the older adults increases with increasing sample size, and the prevalence of obesity in the older adults decreases as the research year increases.

Obesity is a condition in which excessive accumulation of body fat reaches a level that negatively affects a person's health. Factors such as genetic background, high-energy food intake, decreased physical activity, poor diet, gender, age, marital status and monthly income affect the prevalence of obesity. Also, demographic, biological and socio-cultural factors can be considered as effective factors in increasing the prevalence of obesity [69,70].

In a study conducted in the United States, about 1.3 percent of the elderly were with obese and the prevalence of obesity was 27.6 % in men and 33.2 % in women [71]. In another study that looked at 25 provinces in China, the prevalence of obesity in the elderly was 11.53 % [72]. In a study in Australia, the prevalence of this complication in the elderly was reported to be 43.5 % [73]. In an article in India, the prevalence of this complication was 32 % (8) and 3.6 % in a study in South Korea [74]. An article in Mexico also reported a 76 % prevalence of obesity in older women [75]. In a study in Brazil, the prevalence of this complication in the elderly was 32.6 % [76] and in another study, the prevalence of obesity in the elderly in Iran was 30.43 % [77]. A British study found that 32 % of women aged 35 to 64 were overweight and 21 % were with obese, a complication common in women of childbearing age [78].

A study of the elderly in the United States found that high-calorie, high-energy foods that are readily available include reduced levels of physical activity, use of various medications (psychedelics, diabetes, antihypertensive drugs, hormones). Steroids and contraceptives, antihistamines and protease inhibitors) and lack of sleep have increased the prevalence of obesity in this area [79]. Another study in Asia looked at the link between obesity and poverty, saying that in the poorest countries, poverty is associated with malnutrition and underweight, while in middle-income countries, it is associated with an increased risk of obesity [80]. A study in the United Kingdom also found that the United Kingdom had the worst obesity rates of any European country, and that factors such as high-energy foods, socioeconomic status, changes in the environment, reduced sleep hours, and lack of exercise caused Obesity has increased [81]. Also, in a study in Spain explain the different factors in the gender difference in the process of obesity in the elderly. The first factor is physical activity and sedentary lifestyle, which is directly related to obesity at the individual and demographic levels. Spending time sitting, reading or watching TV, sedentary activities, smoking and eating certain foods are other factors [82].

Studies have also been conducted on the association between obesity and Covid 19 disease, which indicate that patients with obesity are at risk of exacerbating viral respiratory infections, but the association between obesity and the severity of the 2019 coronavirus (COVID-19) is unclear [83]. In another study, obesity has been identified as a cause of diabetes and it has been stated that the obesity-related diabetes epidemic is a major crisis in modern societies where food is abundant and exercise is optional [84].

Another article discusses the relationship between obesity, hypertension, and diabetes, which increases the risk of these diseases with increasing obesity [85]. An article also states that both obesity and insulin resistance increase the risk of coronary heart disease, and that insulin resistance at any degree of obesity increases the risk of coronary heart disease and type 2 diabetes [86]. Another study on the elderly in the United States found that body weight has strong effects on metabolic factors that may subsequently affect the risk of cancer, as well as a significant link between obesity and several cancers, such as cancer. There are colon, breast, endometrium, kidney and oesophagus [87]. A study also found that obesity increases maternal and child mortality. Women with obese are more prone to pregnancy complications such as gestational diabetes (GDM) and children of mothers with obese are more prone to cardiovascular and metabolic diseases in adulthood [88].

## 13 Limitations and strength

One of the most important limitations of the present study was that due to the lack of reporting of the prevalence by gender in all studies, the prevalence of obesity in the older adults was not reported by gender, and the most important strength of the present study was the comprehensive review of all databases as well as the heterogeneity based on meta-regression.

## 14 Conclusion

In conclusion, given that the prevalence of obesity in the older adults in the world is high, it is necessary for health policy makers to take effective measures to increase public awareness about the dangers of obesity in the older adults and prevent obesity in the elderly population.

## Ethics approval and consent to participate

Ethics approval was received from the ethics committee of deputy of research and technology, Kermanshah University of Medical Sciences (IR.KUMS.REC.1399.885).

## Consent for publication

Not applicable.

## Availability of data and materials

Datasets are available through the corresponding author upon reasonable request.

## Authors'contributions

AAK and NS and ND and SJ contributed to the design, MM statistical analysis, participated in most of the study steps. SHB and MM and MH prepared the manuscript. All authors have read and approved the content of the manuscript.

## Funding

Funding for this research was provided by the deputy of research and technology –10.13039/501100005317Kermanshah University of Medical Sciences, (4010242), the deputy of research and technology–Kermanshah University of Medical Sciences had no role in this study.

## Declaration of competing interest

The authors declare that they have no known competing financial interests or personal relationships that could have appeared to influence the work reported in this paper.

## Abbreviations

BMI: Body Mass Index
WHR: waist-to-hip ratio
WHtR: waist-to-height ratio
WoS: Web of Science
STROBE: Strengthening the Reporting of Observational studies in Epidemiology
PRISMA: Preferred Reporting Items for Systematic Reviews and Meta-Analysis
